# Age-Related Hypofluorescent Indocyanine Green Angiography Spots: Prevalence, Characteristics, and Associated Features in Age-Related Macular Degeneration

**DOI:** 10.1016/j.xops.2026.101400

**Published:** 2026-09-07

**Authors:** Chui Ming Gemmy Cheung, Zehua Jiang, Noa Gilead, Wataru Kikushima, Christine A. Curcio, Kelvin Yi Chong Teo

**Affiliations:** 1 Medical Retina Department, Singapore Eye Research Institute, Singapore National Eye Centre, Singapore, Singapore; 2 Eye Academic Clinical Program, Duke-NUS Medical School, National University of Singapore, Singapore, Singapore; 3 Graduate Studies, Beijing Visual Science and Translational Eye Research Institute (BERI), Eye Center of Beijing Tsinghua Changgung Hospital, School of Clinical Medicine, Tsinghua Medicine, Tsinghua University, Beijing, China; 4 Department of Ophthalmology and Visual Sciences, University of Alabama at Birmingham School of Medicine, Birmingham, Alabama

**Keywords:** Age-related macular degeneration, Age-related scattered hypofluorescent spots, Indocyanine green angiography, Lipid, Polypoidal choroidal vasculopathy

## Abstract

**Purpose:**

To evaluate the frequency and associated features of detecting age-related scattered hypofluorescent spots (ASHS) on indocyanine green angiography (ICGA) among fellow eyes of patients with unilateral neovascular age-related macular degeneration (nAMD).

**Design:**

Imaging analysis.

**Participants:**

Prospectively recruited cohort with treatment-naive nAMD.

**Methods:**

Patients underwent protocolized examination. Indocyanine green angiography was used to evaluate the presence of ASHS. Choriocapillaris vessel density was determined using OCT angiography (OCTA). The development of incident exudative age-related macular degeneration over 12 months was recorded.

**Main Outcome Measures:**

Prevalence and associated features of ASHS.

**Results:**

We included 142 patients. Macular neovascularization (MNV)-subtype in the fellow eye was polypoidal choroidal vasculopathy (PCV; 60.5%), type I/mixed MNV (26.1%), type II (9.9%), and type III (3.5%). According to ICGA, eyes were categorized into homogeneous (*n* = 48, 33.8%), ASHS (*n* = 46, 32.3%), and drusen/reticular pseudodrusen (RPD) groups (*n* = 48, 33.8%). The ASHS group was significantly younger than the drusen/RPD group (68.4 vs. 75.5 years; *P* < 0.001). The ASHS group had shorter axial length (23.52 vs. 24.32; *P* = 0.047) than the homogeneous group and had better vision (0.17 vs. 0.29; *P* = 0.012) and greater subfoveal choroidal thickness (262.28 vs. 183.04 μm; *P* = 0.006) than the drusen/RPD group. Choriocapillaris vessel density was similar between the ASHS group and the homogeneous group (71.1% vs. 71.2%; *P* = 0.934), but lower in the drusen/RPD group (64.6%; *P* = 0.002). Fellow eye MNV-subtype was predominantly PCV in the ASHS and homogeneous groups (78.26% and 75.00%; *P* = 0.78) but lower in the drusen/RPD group (29.2%; *P* < 0.001). The ASHS group had lower total cholesterol than the homogeneous group (4.31 vs. 4.56mmol/L; *P* = 0.039) and the drusen/RPD group (4.84 mmol/L; *P* = 0.028).

**Conclusions:**

One in 3 fellow eyes of patients with unilateral nAMD in this Asian population exhibited ASHS lesions. Choriocapillaris vessel density based on OCTA was similar between the ASHS and the homogeneous groups, supporting the hypothesis for reduced transit of indocyanine green–laden lipoproteins across the Bruch membrane.

**Financial Disclosure(s):**

Proprietary or commercial disclosure may be found in the Footnotes and Disclosures at the end of this article.

Indocyanine green angiography (ICGA) has been used to delineate areas of neovascularization in neovascular age-related macular degeneration (nAMD) and identify polypoidal variants. These changes typically appear as hyperfluorescent “hot spots” or “plaques” in the mid- to late-phase ICGA. Beyond the area of neovascularization, the fundus appearance shows homogeneous hyperfluorescence in the late-phase ICGA. This appearance is believed to result from retinal pigment epithelium (RPE) uptake of indocyanine green (ICG) dye after it has extravasated into the choroidal stroma and across Bruch membrane (BrM).^1^, ^2^, ^3^, ^4^, ^5^ Disturbance in any part of this transport process of the ICGA dye may result in a hypofluorescent appearance. For example, soft drusen appear hypofluorescent on ICGA, as shown by Arnold et al.^6^ More recently, late-phase hypofluorescent spots in ICGA were observed in eyes without drusen or other corresponding features in either OCT or fluorescein angiography.^7^ Shiraki et al^8^ proposed the term “age-related scattered hypofluorescent spots” (ASHS) on late-phase ICGA (ASHS-LIA) for this observation, although the clinical significance of these spots remains poorly understood.^7^, ^8^, ^9^, ^10^

One proposed explanation is the presence of basal linear deposits (BLinD), a layer of soft drusen material, 0.4 to 2 μm thick between the RPE basal lamina and the inner collagenous layer of BrM.^11^, ^12^, ^13^, ^14^, ^15^, ^16^, ^17^, ^18^, ^19^, ^20^ Both clinically visible drusen and invisible BLinD are composed primarily of low density lipoprotein (LDL)-like lipoproteins made by the RPE, with cores rich in esterified cholesterol. This hydrophobic material can block the transit of ICG across the BrM, thereby resulting in the ASHS-LIA appearance observed in late-phase angiography. It follows that such spots have been associated with normal aging because lipoproteins also accumulate in normal BrM before drusen or BLinD form.

In the present study, we evaluated ICGA performed in patients with unilateral nAMD to evaluate the frequency of detecting scattered hypofluorescent spots in their fellow eyes on late-phase ICGA up to 20 minutes. Because of the difference in timing of late-phase ICGA from previously reported studies on ASHS-LIA, we will refer to these lesions as ASHS in the current work. Additionally, we will compare systemic and ocular features between this group with eyes without this feature and with eyes with drusen. By integrating multimodal imaging, including OCT angiography (OCTA)–derived choriocapillaris (CC) flow deficit and systemic lipid biochemistry, we aim to clarify the structural and metabolic associations of this phenomenon and to explore their role as a biomarker for lipid-related changes at the RPE–BrM interface.

## Methods

Data for this study were taken from fellow eye evaluations of patients recruited into a prospective, observational cohort study (Asian Age-Related Macular Degeneration Phenotyping Study). Detailed methodology has been published elsewhere.^21^ The study was approved by the Singhealth Centralized Institutional Review Board (R697/47/2009) and was conducted according to the tenets of the Declaration of Helsinki. Written informed consent was obtained from each patient before participation in the study. Data for this study were taken from patients recruited between October 2019 and 2023 at the Singapore National Eye Centre.

Briefly, the Asian Age-Related Macular Degeneration Phenotyping Study prospectively recruited consecutive treatment-naive participants with exudative nAMD from the retinal clinic of the Singapore National Eye Centre beginning in March 2010 and is still ongoing. Basic demographic and clinical information were collected by trained study coordinators, and all imaging examinations were performed following standardized acquisition protocols. All patient data were fully deidentified before analysis. Each patient was examined at baseline according to a protocol including a standardized history, clinical examination including measurement of best-corrected visual acuity, slitlamp biomicroscopy, indirect fundus examination, and color fundus photos (TRC-50DX [type IA]; Topcon). Fluorescein angiography, ICGA, and spectral-domain OCT with enhanced depth imaging mode were performed using the Heidelberg Spectralis (Heidelberg Engineering). Thirty degrees of the retina were scanned along the horizontal plane through the foveal center. A minimum signal strength of 20 was required. Nine images were averaged for each scan. Swept-source OCTA en face images (PlexElite, Zeiss) were used to evaluate CC vessel density.

### Medical and Drug History

A detailed interviewer-administered questionnaire was used to collect information about medical history (including hypertension, diabetes, angina, myocardial infarction, and stroke), medication, including lipid-altering drugs, and cigarette smoking (defined as current, past, and never) in participants of both studies. A nonfasting venous blood sample was collected at baseline. Lipid biochemistry was performed using standard automated methods in the Biochemistry Department at the Singapore General Hospital, with a Beckman Coulter ExC800 automated chemistry analyzer. Total cholesterol, high density lipoprotein cholesterol, triglycerides, and LDL cholesterol were analyzed based via homogeneous enzymatic colorimetry assay using 500 μL of serum.

### Follow-up and Incident Exudative Neovascularization

All patients had a minimum of 12 months of follow-up. During follow-up, incident exudation confirmed to result from new neovascularization was recorded.

### Image Grading

For this analysis, only fellow eyes without active choroidal neovascularization, geographic atrophy, scarring, central serous chorioretinopathy, or other macular pathology affecting ICGA interpretation were included. Image evaluations were independently reviewed by 2 graders (Z.J. and N.G.) and confirmed by a third senior retinal specialist in cases of disagreement (C.M.G.C.). Late-phase ICGA images (taken ≥10 minutes after dye injection) were used to evaluate the presence of scattered hypofluorescent spots and choroidal vascular hyperpermeability.

Based on ICGA, eyes were divided into following 2 broad categories: a homogeneous group, defined by a uniform homogeneous ICGA background appearance, and a nonhomogeneous group, which showed focal abnormalities. The nonhomogeneous group was further subdivided using multimodal imaging into ASHS group and the drusen/reticular pseudodrusen (RPD) group. Age-related scattered hypofluorescent spots was defined by the presence of hypofluorescent spots with a minimum diameter of 100 μm in diameter on ICGA, visible not before 10 minutes after dye injection, showing no corresponding abnormalities on OCT, color fundus photos, and fluorescein angiography. Eyes with focal RPE-BrM thickening, subretinal drusenoid deposits on OCT, and corresponding visible drusen of any type were categorized to the drusen group.

The drusen or RPD group was defined by focal hypofluorescent lesions in late-phase ICGA corresponding to soft drusen or RPD on color fundus photography and OCT (Fig 1). The subtype of neovascularization in the first eye was evaluated based on multimodal imaging. The presence of polypoidal choroidal vasculopathy (PCV) was noted based on criteria used in the EVEREST (efficacy and safety of verteporfin photodynamic therapy in combination with ranibizumab or alone versus ranibizumab monotherapy in patients with symptomatic macular polypoidal choroidal vasculopathy) study.^22^ Subfoveal choroidal thickness (CT) was measured using the built-in measuring tool of the Heidelberg Eye Explorer software (Heidelberg Engineering). Choroidal vascular hyperpermeability was characterized by multifocal choroidal hyperfluorescence with blurred margins and dilated hyperpermeable choroidal blood vessels.^23^

**Figure 1:**
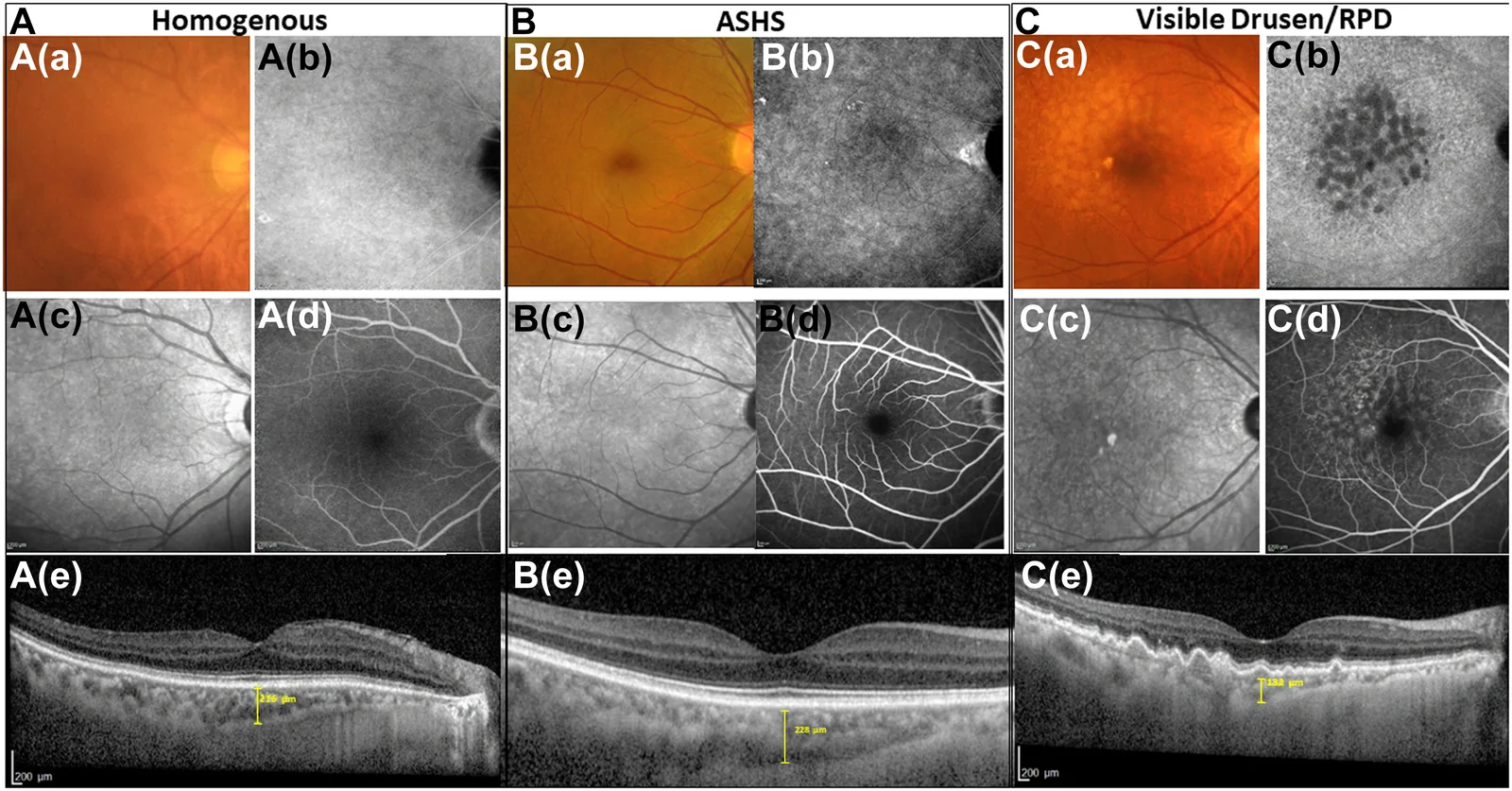
**A,** Examples of multimodal imaging findings in eyes with homogeneous late-phase indocyanine green angiography. **B,** Age-related scattered hypofluorescent spots (ASHS). **C,** Eyes with drusen/reticular pseudodrusen (RPD). For each example, we display color fundus photography (a), late-phase indocyanine green angiography (12–20 minutes after injection) (b), near-infrared (c), late-phase fluorescein angiography (≥5 minutes after injection) (d), and OCT (e).

From September 2022, 6 × 6 mm^2^ OCTA scans of the macular region centered on the fovea were acquired using PlexElite OCTA (Zeiss), which was incorporated into the protocol to allow visualization of the CC. A CC slab was automatically segmented using the built-in algorithm, which defined the region extending from BrM to 20 μm into the inner choroid, to visualize the flow signal within the CC. Manual verification and correction of segmentation errors were performed where appropriate by graders masked to the ICGA findings.^24^ The resulting en face images of the CC were exported and processed in ImageJ (version 1.54p; National Institutes of Health). Images affected by artifacts or poor quality were excluded from further quantitative evaluation. To reduce projection artifacts and speckle noise, the en face structural CC image first underwent inverse transformation and Gaussian smoothing using a 3 × 3 pixel kernel. The processed structural image was then multiplied with the corresponding en face CC flow image. The resulting images were subsequently converted to 8-bit grayscale before binarization. Local thresholding was performed using the Phansalkar method with a radius of 4 pixels, generating binary masks in which flow-positive regions appeared as white pixels.^25^^,^^26^ Pixel counts were extracted from the image histogram, and CC vascular density was calculated as the proportion of white pixels relative to the total image pixel count.^25^^,^^27^

### Statistical Analysis

Basic demographic and clinical information—including age, sex, race, general parameters (height, weight, body mass index, smoking history, and use of lipid-lowering medications), blood pressure parameters (systolic pressure, diastolic pressure, and pulse rate), laboratory values (hemoglobin A1c, total cholesterol, high density lipoprotein-C, LDL-C, triglycerides, and C-reactive protein), and ocular parameters (eye laterality, lens status, distance visual acuity, intraocular pressure, axial length, and structural metrics such as central subfield thickness, subfoveal CT, OCTA en face CC density, and choroidal vascular hyperpermeability)—were summarized. The type of choroidal neovascularization in the diseased eye was also recorded. All variables were included in further correlation and intergroup comparison analyses. These comparisons were performed to characterize the predefined groups using clinically relevant systemic and ocular variables. Because the analyses were descriptive in nature, no formal adjustment for multiple comparisons was applied.

Statistical analyses were performed using IBM SPSS Statistics version 25.0 (IBM Corp) and R version 4.3.3 (February 29, 2024; R Foundation for Statistical Computing). Continuous variables were summarized as mean ± standard deviation or median (interquartile range) and compared using the Student *t* test or Kruskal–Wallis test, as appropriate. Categorical variables were presented as frequencies (percentages, %) and compared using the χ^2^ test. Group comparisons were adjusted for age using analysis of covariance for continuous variables and logistic regression for categorical variables if needed. Univariate logistic regression analyses were first conducted to explore associations between candidate variables and ASHS. Variables with a *P* value <0.05 in univariate analysis were subsequently included in multivariate logistic regression models to identify independent factors. Odds ratios and 95% confidence intervals were reported. All 2-tailed *P* values <0.05 were considered statistically significant. The log-rank test was used to compare the incidence of neovascularization over follow-up.

## Results

We identified 267 patients with ICGA during the study period. Eyes with inadequate imaging and those with obvious pathologies (e.g., geographic atrophy, neovascularization, and central serous chorioretinopathy) in the fellow eye were excluded. We also excluded 5 eyes with extensive cuticular drusen because they could interfere with ICGA grading. A further 7 eyes with both soft drusen and ASHS were excluded from the analysis. Among the remaining 142 eyes from 142 patients that were included, the mean age was 70.8 years (standard deviation = 8.2), with 70.4% being male and 90.1% identified as Chinese ethnicity. The subtype of macular neovascularization (MNV) in the fellow eye was PCV (60.5%), type I or mixed-type MNV (26.1%), pure type II MNV (9.9%), and type III MNV (3.5%).

Based on ICGA, the study eyes were classified into 3 groups: the homogeneous (*n* = 48, 33.8%), the ASHS (*n* = 46, 32.3%), and the drusen/RPD groups (*n* = 48, 33.8%) (Fig 1). Within the ASHS group, the ASHS lesions were distributed within the central 3 mm diameter of the ETDRS grid in 52.2% (*n* = 24) and extended beyond the central 3 mm in 47.8% (*n* = 22). Within the drusen/RPD group, 26 had soft drusen only, 2 had RPD only, and 19 had both soft drusen and RPD. The hypofluorescent lesions corresponding to the drusen were readily detectable in the early phase ICGA and persisted through the mid- and late-phase. Additionally, pachydrusen were present in 17 eyes (10 in the homogeneous group and 7 in the ASHS group). The pachydrusen appeared either hyperfluorescent (*n* = 15) or isofluorescent (*n* = 2) and did not interfere with the interpretation of ASHS because they were primarily outside the vascular arcades, were few in number, and were small in size. OCT angiography was available in 48 patients since September 2022 onward (homogeneous, *n* = 14/48; ASHS, *n* = 17/46; drusen/RPD, *n* = 18/48).

### Comparison between the ASHS versus Homogeneous Groups

The ASHS group had similar age, sex, ethnicity, and medical history as patients in the homogeneous group (Table 1). Regarding lipid profiles, the ASHS group had significantly lower total cholesterol (4.31 [1.10] vs. 4.56 [1.48 mmol/L]; *P* = 0.039) and triglyceride (1.28 [0.68] vs. 1.61 [1.15]; *P* < 0.001) than the homogeneous group after adjusting for lipid-lowering medication use. Regarding ocular features, the ASHS group had shorter axial length (23.52 ± 1.28 vs. 24.32 ± 1.49; *P* = 0.047). Choriocapillaris vessel density was similar between the 2 groups (71.1 ± 2.9% vs. 71.2 ± 4.3%; *P* = 0.934) (Fig 2). The subtype of neovascularization in the fellow eye was predominantly PCV (75.00% and 78.26%; *P* = 0.78) in both groups. The incidence of neovascularization in study eyes over a mean follow-up of 3.42 ± 2.02 years was similarly low (4.08% and 0.00%) over the follow-up period in the homogeneous and the ASHS groups, respectively (*P* = 0.94). All incident cases of neovascularization in the homogeneous group (*n* = 2) were of the PCV subtype.

**Table 1:** Comparisons of Systemic and Ocular Features between the Homogeneous and ASHS Groups and between the ASHS and Drusen Groups

	Homogeneous,∗ Mean ± SD or IQR/*N* (%)	ASHS,† Mean ± SD/*N* (%)	*P*∗ vs. †	Drusen,‡ Mean ± SD/*N* (%)	*P*∗ vs. ‡
Number	*N* = 48	*N* = 46		*N* = 48	
Age, yrs	68.27 ± 6.94	68.39 ± 7.30	0.935	75.52 ± 8.37	**<0.001**
Sex, male	34 (70.83)	34 (73.91)	0.738	32 (66.67)	0.122
Races			0.563		0.502
Chinese	42 (87.50)	41 (89.13)		45 (93.75)	
Malay	4 (8.33)	2 (4.35)		3 (6.25)	
Indian	2 (4.17)	1 (2.17)		0 (0.00)	
Japanese	0 (0.00)	1 (2.17)		0 (0.00)	
Filipino	0 (0.00)	1 (2.17)		0 (0.00)	
Personal history					
Smoking	16 (33.33)	13 (28.26)	0.588	20 (41.67)	0.286
Diabetes	17 (35.42)	14 (30.43)	0.596	17 (35.42)	0.954
Hypertension	38 (80.85)	34 (73.91)	0.420	32 (66.67)	0.121
Hyperlipidemia	31 (64.58)	30 (65.22)	0.957	24 (50.00)	0.054
Cardiovascular disease	2 (4.17)	4 (8.70)	0.370	3 (6.25)	0.698
Stroke	0 (0.00)	3 (6.52)	0.999	0 (0.00)	0.999
Lipid-lowering medication	27 (56.25)	26 (56.52)	0.982	14 (29.17)	**0.009**
Body mass index	25.27 ± 3.31	25.31 ± 4.93	0.991	23.59 ± 4.20	0.420
Blood pressure indexes, *N*	*N* = 27	*N* = 29		*N* = 33	
Systolic pressure, mmHg	144.93 ± 18.44	143.00 ± 17.45	0.450	146.27 ± 21.50	0.920
Diastolic pressure, mmHg	78.46 ± 10.34	73.43 ± 10.88	0.787	64.73 ± 15.68	0.591
Pulse rate	70.54 ± 13.72	67.40 ± 9.02	0.639	64.73 ± 11.55	0.525
Laboratory indexes, *N*	*N* = 32	*N* = 32	*P*§	*N* = 36	*P*§
Glucose level, fasting	6.88 ± 2.15	6.46 ± 1.06	0.651	7.00 ± 2.28	0.769
HbA1c	6.52 ± 1.14	6.46 ± 0.89	0.842	6.17 ± 0.78	**0.049**
Cholesterol	4.56 (1.48)	4.31 (1.10)	**0.039**	4.84 (1.63)	**0.028**
HDL cholesterol	1.30 (0.41)	1.23 (0.45)	0.772	1.33 (0.61)	0.558
LDL cholesterol	2.44 (1.25)	2.30 (0.88)	0.084	2.68 (1.37)	0.407
Triglycerides	1.61 (1.15)	1.28 (0.68)	**0.002**	1.27 (0.87)	0.869
Creatine, serum	91.57 ± 15.32	88.50 ± 44.40	0.905	91.09 ± 29.90	0.346
C-reactive protein	9.76 ± 28.76	5.34 ± 7.87	0.745	7.79 ± 15.55	0.919
Ocular indexes					
Eyes, right	23 (47.92)	21 (45.65)	0.996	27 (56.25)	0.533
Lens status, phakic	32 (66.67)	36 (78.26)	0.194	28 (58.33)	0.644
Distance visual acuity, LogMAR	0.21 ± 0.40	0.17 ± 0.13	0.529	0.29 ± 0.24	**0.012**
Intraocular pressure, mmHg	16.48 ± 3.53	16.35 ± 3.62	0.741	15.79 ± 2.97	0.912
Axial length, mm	24.37 ± 1.49	23.52 ± 1.28	**0.037**	23.99 ± 1.07	0.163
OCT					
Central subfield thickness, μm	264.54 ± 32.74	262.59 ± 24.95	0.508	260.00 ± 29.31	0.554
Subfoveal choroidal thickness, μm	227.83 ± 106.20	262.28 ± 98.64	0.080	183.04 ± 80.88	**0.006**
ICGA					
Choroidal vascular hyperpermeability	27 (56.25)	21 (45.65)	0.301	27 (56.25)	0.228
MNV subtype in first eye			0.537		**<0.001**
PCV	36 (75.00)	36 (78.26)		14 (29.17)	
Type I CNV	6 (12.50)	6 (13.04)		19 (39.58)	
Type II CNV	5 (10.42)	4 (8.70)		5 (10.42)	
Mixed-type I and II CNV	1 (2.08)	0 (0.00)		5 (10.42)	
Type III CNV	0 (0.00)	0 (0.00)		5 (10.42)	
OCTA	*N* = 13	*N* = 17		*N* = 18	
En face choriocapillaris density, %	71.1 ± 2.9	71.2 ± 4.3	0.934	64.6 ± 6.8	**0.002**
Incident neovascularization during follow-up	2 (4.08)	0 (0.00)	*P*‖ 0.940	11 (22.92)	*P*‖**0.001**

ASHS = age-related scattered hypofluorescent spots; CNV = choroidal neovascularization; HbA1c = hemoglobin A1c; HDL = Xhigh density lipoprotein, ICGA = indocyanine green angiography; IQR = interquartile range; LDL = low density lipoprotein; LogMAR = logarithm of the minimum angle of resolution; MNV = macular neovascularization; OCTA = OCT angiography; PCV = polypoidal choroidal vasculopathy; SD = standard deviation.

Group comparisons were adjusted for age using analysis of covariance for continuous variables and logistic regression for categorical variables, unless otherwise specified.

Bold font indicates *P* < 0.05.

∗ Homogenous group.

† ASHS group.

‡ Drusen/RPD group.

§ Comparisons of serum lipid variables (total cholesterol, HDL cholesterol, LDL cholesterol, and triglycerides) were adjusted for both age and lipid-lowering medication use using analysis of covariance.

‖ Comparison of cumulative incidence was performed using the log-rank test.

**Figure 2:**
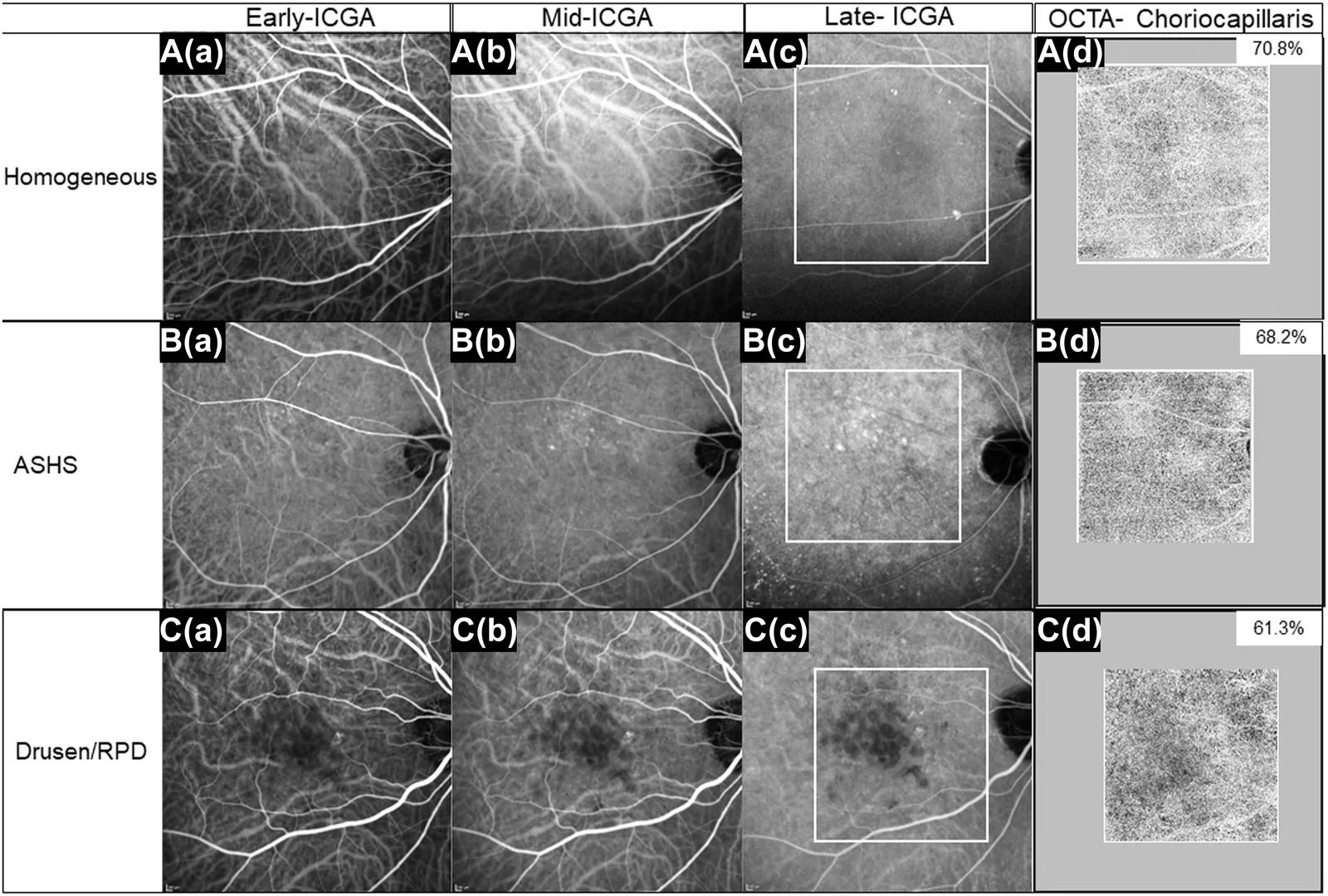
**A,** Examples of indocyanine green angiography (ICGA) and OCT angiography (OCTA) of the choriocapillaris in eyes with homogeneous late-phase ICGA. **B,** Age-related hypofluorescent spots. **C,** Eyes with drusen/reticular pseudodrusen (RPD). For each example, we display early phase ICGA (a), mid-phase ICGA (b), late-phase ICGA (c), and the 6- × 6-mm OCTA choriocapillaris slab (d) centered over the fovea. The inset in panel (d) shows the vessel density of the choriocapillaris in the scanned area. ASHS = age-related scattered hypofluorescent spots.

### Comparison between the ASHS and Drusen/RPD Groups

The ASHS group was significantly younger than the drusen/RPD group (68.4 ± 7.3 vs. 75.5 ± 8.4 years; *P* < 0.001) (Table 1). Regarding lipid profiles, a higher proportion of patients in the ASHS group were receiving lipid-lowering medication (56.5% vs. 29.2%; *P* = 0.009). After adjusting for age and lipid-lowering medication, the ASHS group still had significantly lower total cholesterol levels (4.31 ± 1.10 vs. 4.84 ± 1.60 mmol/L; *P* = 0.028) than the drusen/RPD group. At the ocular level, the ASHS group had better visual acuity (0.17 ± 0.13 vs. 0.29 ± 0.24 logarithm of the minimum angle of resolution; *P* = 0.012) and greater subfoveal CT (262.28 ± 98.64 vs. 183.04 ± 80.88 μm; *P* = 0.006). The subtype of neovascularization in the fellow eye was more frequently PCV in the ASHS group (78.3% vs. 29.2%; *P* < 0.001). Choriocapillaris vessel density was significantly higher in the ASHS group than in the drusen/RPD group (71.2 ± 4.3% vs. 64.6 ± 6.8%; *P* = 0.002) (Fig 2). The incidence of exudative MNV in study eyes was lower in the ASHS group than in the drusen/RPD group (0.0% vs. 22.9%; *P* < 0.001), over a similar follow-up duration (3.00 ± 1.87 vs. 3.35 ± 1.80 years). Among the 11 incident cases of neovascularization in the drusen/RPD group, type I MNV was most frequent (n = 5), followed by PCV (n = 3) and type III MNV (n = 3).

## Discussion

We report that 1 in 3 fellow eyes of patients with unilateral nAMD in this Asian population exhibited ASHS lesions in late-frame ICGA taken ≥10 minutes after dye injection. These ASHS lesions are not apparent before 10 minutes, have a minimum diameter of 100 μm, and had no abnormalities in the corresponding color fundus photos, OCT, or fluorescein angiography images. Approximately half of the eyes had ASHS lesions beyond the central 3-mm diameter of the ETDRS grid when we evaluated the 30° imaging field of view. We believe these ASHS lesions represent similar entities that previous researchers have described as ASHS-LIA.^7^, ^8^, ^9^, ^10^ However, because our imaging protocol differs from previous studies in the timing of the late-phase ICGA frames used (12–20 vs. >30 minutes) and the imaging field of view (30° centered over the fovea vs. up to 55° or wider), we have refrained from using the term ASHS-LIA for our observation.

A significant novel observation in the current study is that CC vessel density based on OCTA was similar between the ASHS and the homogeneous groups, suggesting that CC perfusion insufficiency is not a key contributor to the appearance of ASHS (Table 2). Together with the previous important observation using adaptive optics–enhanced ICG, which observed that RPE structure and density were not affected within ASHS lesions, the interpretation of ASHS can now focus on reduced transit of ICG-laden lipoproteins across the BrM as the most likely mechanism.^28^

**Table 2:** Summary of Characteristics of the ASHS Group Compared with the Homogeneous and the Drusen/RPD Groups

	Characteristics of the ASHS group	
	Compared with Homogeneous	Compared with Drusen/RPD
Age	NS	Younger
Sex	NS	NS
Lipid profile	Lower cholesterol and triglyceride	Lower cholesterol
Ocular features	Shorter axial length	Better vision Thicker subfoveal choroid
Choriocapillaris vessel density	NS	Higher
MNV incidence	NS	Lower
Fellow eye MNV subtype	Predominantly PCV in both	More frequently PCV

ASHS = age-related scattered hypofluorescent spots; MNV = macular neovascularization; NS = not significant; PCV = polypoidal choroidal vasculopathy; RPD = reticular pseudodrusen.

The late-phase ICGA appearance can be interpreted by considering the sequential movement of ICG dye from the circulation to the RPE. In normal eyes, dye extravasates from the CC, traverses BrM, and is then taken up by the RPE, resulting in a homogeneously hyperfluorescent background in late frames.^1^^,^^2^^,^^5^ Abnormal hypofluorescence in the late-phase ICGA therefore may arise from disruption at ≥1 of these levels. Reduced availability of ICG-laden lipoproteins at the RPE may occur in compromised CC endothelial transport. In such cases, hypofluorescence typically appears at the early phases of ICGA and persists throughout, reflecting endothelial failure. A second possibility is impaired transport across the BrM. With aging and age-related macular degeneration, the BrM develops a “lipid wall” composed of RPE-derived, esterified cholesterol-rich deposits (BLinD).^11^^,^^29^^,^^30^ Finally, reduced internalization of ICG dye may result from RPE dysfunction or dysregulation of lipoprotein receptors. Uptake of lipid-soluble nutrients such as macular xanthophyll carotenoids relies on membrane-bound class B scavenger receptors and LDL receptors at the RPE.^31^, ^32^, ^33^, ^34^ Loss or altered function of these transporters could impair ICG-laden lipoprotein uptake.

The BLinD may hinder the passage of macromolecules such as ICG–lipoprotein complexes, whereas smaller particles such as plasma high density lipoprotein (11 nm) may still cross. Structural remodeling of BrM, including increased collagen cross-linking,^35^ deposition of advanced glycation end products,^36^ and proteoglycan sulfation, increases stickiness for retaining lipoproteins. These barrier properties may collectively limit dye availability at the RPE.^37^ Studying late-phase ICGA has been proposed to be a potential imaging biomarker for BLinD. In the limited previous work around late-phase ICGA, Shiraki et al^8^ used video ICGA in 91 eyes from individuals aged ≥50 years without soft drusen and coined the term ASHS-LIA, which was present in ∼20% of eyes. These authors did not discuss the possible origin of the feature.^8^ Subsequently, Chen et al^7^^,^^9^^,^^38^ used confocal scanning laser ophthalmoscope ICGA to evaluate ASHS-LIA in aging and age-related macular degeneration and proposed BLinD to be the reason for the hypofluorescence observed. Our results are consistent with these previous reports in the appearance of the spots, which are 100 to 500 μm in diameter and may be distributed within the central 3 mm or more diffusely over the ETDRS 6-mm area. The ocular associations with PCV and CT were also similar.

We observed lower cholesterol and lower triglyceride levels in the ASHS group than in the homogeneous group, after adjusting for age, sex, and lipid-lowering medication. However, whether systemic lipid profile reflects the local microenvironment remains a subject of debate.^39^, ^40^, ^41^, ^42^ Systemic lipid levels may not directly reflect lipid accumulation or lipoprotein handling at the RPE–BrM interface. In addition, conventional lipid measures may not capture changes such as alterations in lipoprotein particles, which may be more reflective of their cholesterol transport capacity.^43^

Our original hypothesis was that ASHS and visible drusen represent stages of BLinD within a continuous spectrum of impaired transport within the BrM. However, significant systemic and ocular differences were observed at baseline. The drusen/RPD group was significantly older, less likely to have been diagnosed with hyperlipidemia or take lipid-lowering medications, had higher cholesterol levels, and had significantly thinner choroid compared with the ASHS group. The subtype of MNV in the first eye was also different, with fewer patients in the drusen/RPD group having PCV in their first eye. The drusen/RPD group also had a significantly higher rate of incident neovascularization than both the homogeneous and the ASHS groups (22.92% vs. 4.08% vs. 0.00%; *P* = 0.001). Furthermore, only 7 eyes demonstrated concurrent ASHS and visible drusen and were excluded from the primary analysis because of their small number (Table S1, available at www.ophthalmologyscience.org). Importantly, none of the eyes in the ASHS group developed visible drusen within the follow-up period. Although no formal comparisons were performed, the excluded eyes appeared to have relatively lower serum cholesterol and triglyceride levels, consistent with the overall association between ASHS and altered systemic lipid profiles observed in the primary analyses. Larger studies are needed to better characterize this subgroup and determine its clinical significance. Therefore, despite similarity in impaired transport across BrM, additional factors may determine the development of visible drusen. Interestingly, we have previously reported that serum from patients with PCV has higher cholesterol efflux capacity than that of those with typical nAMD.^44^

Strengths of the current study include several novel aspects: the evaluation of the CC with OCTA, prospective follow-up, incident neovascularization rates, and evaluation of systemic lipid biochemistry. Limitations should also be mentioned. First, the late-phase images in the present study were taken between 15 and 20 minutes, whereas previous studies reporting ASHS-LIA have included ICGA taken at 30 minutes after injection. The residual fluorescence from dye within the choroidal circulation at the earlier timepoint could interfere with detection of ASHS. Second, the limited field of view restricts the ability to detect changes beyond the central area. Together, these differences may contribute to the lower prevalence of ASHS in the current series than that reported in other series.^3^^,^^38^ Lipid profiles were obtained from nonfasting blood samples, which may affect triglyceride measurements. However, previous studies have suggested that the impact of fasting may be limited.^45^, ^46^, ^47^ OCT angiography was only available in a subset of eyes after we acquired the PlexElite system in 2022. After this date, consecutive patients were included; hence, they were fairly well represented across groups and were not subjected to any selection. Finally, the group with concurrent drusen and ASHS was excluded from the analysis because of the very small sample size of only 7 eyes. Another consideration is that grading of ASHS can be subjective. Future efforts to standardize its diagnostic threshold and harmonize intergrader experience are important.

In conclusion, we observed ASHS lesions in a notable proportion of fellow eyes of patients with unilateral nAMD. By incorporating OCTA-derived CC density measurements, we further distinguished these lesions from perfusion defects and reinforced mechanisms centered on impaired transport across BrM. Although experiments have evaluated the lipoproteins responsible for transporting ICG dye in mice,^48^ future studies should aim to elucidate the detailed lipoprotein subfraction(s) responsible for transporting ICG dye into the RPE in humans.
