# Supplementary material for: Age-Related Hypofluorescent Indocyanine Green Angiography Spots: Prevalence, Characteristics, and Associated Features in Age-Related Macular Degeneration
Source: Ophthalmol Sci. 2026 Sep 7;6(11):101400. doi: 10.1016/j.xops.2026.101400 (PMC13673339; doi:10.1016/j.xops.2026.101400)
Supplement: Supplementary file 1 [file mmc1.pdf]

**Table S1. Clinical characteristics of the seven excluded eyes with both drusen and Hypospots**

| Characteristic | Homogenous (n=48) | ASHS (n=46) | Drusen (n=48) | ASHS & Drusen* (n=7) |
|---|---|---|---|---|
| Age, years | 68.3 ± 6.9 | 68.4 ± 7.3 | 75.5 ± 8.4 | 68.3 ± 4.4 |
| Male sex, n (%) | 34 (70.8%) | 34 (73.9%) | 32 (66.7%) | 7 (100%) |
| Distance visual acuity, LogMAR | 0.21 ± 0.40 | 0.17 ± 0.13 | 0.29 ± 0.24 | 0.23 ± 0.19 |
| PCV in fellow eye, n (%) | 36 (75.0%) | 36 (78.3%) | 14 (29.2%) | 5 (71.4%) |
| Type 1/mixed MNV in fellow eye, n (%) | 6 (12.5%) | 6 (13.0%) | 19 (39.6%) | 2 (28.6%) |
| Total cholesterol (mmol/L)† | 4.56 ± 1.48 | 4.31 ± 1.10 | 4.84 ± 1.60 | 4.10 ± 0.79 |
| HDL-C (mmol/L)† | 1.33 ± 0.37 | 1.36 ± 0.37 | 1.44 ± 0.43 | 1.36 ± 0.15 |
| LDL-C (mmol/L)† | 2.89 ± 1.27 | 2.43 ± 0.72 | 2.81 ± 1.22 | 2.23 ± 0.84 |
| Triglycerides (mmol/L)† | 1.61 ± 1.15 | 1.28 ± 0.68 | 1.40 ± 0.72 | 1.13 ± 0.24 |
| Subfoveal choroidal thickness (μm) | 227.8 ± 106.2 | 262.3 ± 98.6 | 183.0 ± 80.9 | 286.4 ± 141.9 |

* Eyes demonstrating both Age-related Hypofluorescent spots (ASHS) and drusen that were excluded from the primary analyses because of their small number.
